# CPNE1 is a potential prognostic biomarker, associated with immune infiltrates and promotes progression of hepatocellular carcinoma

**DOI:** 10.1186/s12935-022-02485-2

**Published:** 2022-02-09

**Authors:** Jinfang Su, Yongbiao Huang, Yali Wang, Rui Li, Wanjun Deng, Hao Zhang, Huihua Xiong

**Affiliations:** Department of Oncology, Tongji Hospital, Tongji Medical College, Huazhong University of Science and Technology, Wuhan, Hubei China

**Keywords:** Hepatocellular carcinoma, CPNE1, Prognosis, AKT/P53, Immune infiltration

## Abstract

**Background:**

Copine1 (CPNE1), the first discovered CPNE1 family member, participates in the process of carcinogenesis and development of diverse tumors. Our study aimed to investigate the expression and prognostic value of CPNE1 gene in hepatocellular carcinoma (HCC), to explore its functional network in HCC and its effects on biological behaviors.

**Methods:**

HCCDB, CCLE, HPA and LinkedOmics online databases were used to explore the expression of CPNE1 gene and analyze the co-expression network of CPNE1 in hepatocellular carcinoma. Gene set enrichment analysis (GSEA) was used for GO functional annotation, KEGG pathway enrichment analysis and regulators of CPNE1 networks in LIHC. HepG2 and MHCC-97H cells were selected to construct CPNE1 knockdown cell lines by transfection with siRNA, and Hep3B cell was selected to construct CPNE1 overexpression cell line by transfection with plasmid. The effect of CPNE1 on the proliferation of hepatocellular carcinoma cells was examined by CCK8 assay and clone formation assay; the effect of CPNE1 on the migration ability of hepatocellular carcinoma cells was assessed by cell scratch assay and Transwell cell migration assay; finally, the expression of related signaling pathway proteins was examined by Western Blot. The correlation of CPNE1 expression with immune infiltration and immune checkpoint molecules in HCC tissues was analyzed using TIMER online database and GSEA.

**Results:**

CPNE1 was highly expressed in HCC tissues and significantly correlated with sex, age, cancer stage and tumor grade. Overall survival (OS) was significantly lower in patients with high CPNE1 expression than in patients with low CPNE1 expression, and CPNE1 could be used as an independent prognostic indicator for HCC. Knockdown of CPNE1 gene inhibited the AKT/P53 pathway, resulting in decreased proliferation, migration and invasion of HCC cells. Overexpression of CPNE1 gene showed the opposite results. The level of CPNE1 expression in HCC was significantly and positively correlated with the level of infiltration of B cells, CD8^+^ T cells, CD4^+^ T cells, macrophages, neutrophils, and dendritic cells (P < 0.001). GSEA results also showed that CPNE1 of LIHC was involved in some immune response regulating signaling pathways.

**Conclusions:**

Our study firstly found the expression of CPNE1 was significantly higher in LIHC tissues than in normal liver tissues, and high CPNE1 expression was associated with poor prognosis. In addition, we identified the possible mechanism by which CPNE1 functioned in LIHC. CPNE1 influenced AKT/P53 pathway activation and LIHC cell proliferation and migration. There was a significant correlation between CPNE1 expression and tumor immune infiltration in LIHC.

**Supplementary Information:**

The online version contains supplementary material available at 10.1186/s12935-022-02485-2.

## Introduction

Liver cancer, one of the top five deadliest cancers globally, has the high mortality [1, 2]. Liver hepatocellular carcinoma (LIHC), the major histology subtype of primary liver cancer, accounts for 70–80% proportion of total liver cancer cases and is chiefly related to hepatitis C virus (HCV), hepatitis B virus (HBV) and alcoholism [3, 4]. Surgical resection or liver transplantations is the common treatment choice in patients with early hepatocellular carcinoma. However, many cases are usually refractory to treat surgically due to initial diagnosis at an advanced stage. Although advanced LIHC exists multiple treatments, including surgical excision, transarterial embolization, chemotherapy and radiofrequency ablation, the treatments effects are limited and LIHC still has a rate of recurrence as high as 70% [5, 6]. In brief, patients with LIHC have a poor overall survival. Despite the risk factors (HBV, HCV, alcohol-related cirrhosis, smoking, diabetes, fatty liver disease, obesity, iron overload and multiple diet exposure) of LIHC is well known, the precise mechanism underlying development and progression of LIHC remains unclear [7, 8]. Therefore, in-depth studies exploring novel biomarkers and delineating its mechanism are urgently needed.

Copines family, a widely distributed and highly conserved throughout evolution phospholipid‐binding protein, shares common structural features: 2 C2 domains in the N terminus, 1 von Willebrand factor A (VWA) domain in the C terminus [9, 10]. C2 domains possess properties of Ca2+ dependence and phospholipid-binding and may be associated with signal transduction and cell membranes transport. VWA domain could mediate interactions among extracellular proteins and may be related to recruitment of target proteins and regulating activity of specific proteins [11, 12]. In mammals, it has been identified that Copines family contains 9 members named sequentially as CPNE1 ~ 9 in order of discovery [10].

Copine1 (CPNE1), the first discovered CPNE1 family member [9], is located on human chromosome 20q11.21, encodes 537 amino acids and has multiple splice forms [13]. CPNE1 is observed to be upregulated in multiple tumor tissue compared to normal tissues. Studies have highlighted that CPNE1 involves in various cellular biology process, such as apoptosis, growth control, autophagy, mitotic, inflammation, exocytosis and cytoskeletal organization and gene transcription [14]. Meanwhile, CPNE1 participates in the process of carcinogenesis and development of breast cancer [15], non‐small cell lung cancer [16], prostate cancer [17], liver cancer [18], thyroid cancer [19] and osteosarcoma [20]. The expression of CPNE1 is associated with TNM staging, lymph node metastasis and distant metastasis of lung adenocarcinoma [16]. The expression of CPNE1 is higher in prostate cancer tissue and castration-resistant prostate cancer tissue than that in normal prostatic tissues and noncastrated-resistant prostate cancer tissue, respectively. Also, CPNE1 is significantly correlated with the tumor stage, Gleason score and recurrence-free survival of prostate cancer and is positively correlated with expression of TRAF2 as a prognostic marker in prostate cancer [17]. CPNE1 is linked to chromosome deletion of 13q in hepatic carcinoma cells and mediates the process of occurrence and progression by regulating the dedifferentiation, cell cycle and proliferation in liver cancer [18]. CPNE1 can act as potential biomarker to identify well-differentiated thyroid cancer tissue and normal thyroid tissues, which simplifies the process of early thyroid cancer diagnosis [19].

Previous studies have uncovered the value of CPNE1 in multiple cancers, yet, the exact role and latent mechanism of CPNE1 in LIHC are unclear. In the current study, we deeply explored the effect of CPNE1 on biological behaviors of LIHC cell lines through a variety of ways. We found that the AKT/P53 signaling was linked in the abilities of CPNE1 to stimulate the proliferation and cell migration in LIHC cells.

## Methods

### Expression analysis and survival analysis

We searched for the gene symbol 'CPNE1' using the HCCDB database. HCCDB provides visualization of the results of multiple computational analyses, such as differential expression analysis, tissue-specific and tumor-specific expression analysis [21]. Then, the expression of CPNE1 in cancer cell lines was validated using the Cancer Cell Line Encyclopedia (CCLE) dataset (https://portals.broadinstitute.org/ccle) [22]. In addition, we validated the protein expression of CPNE1 in the Human Protein Atlas (HPA) database (www.proteinatlas.org) [23].

The UALCAN database (http://ualcan.path.uab.edu) [24] was used for subgroup analysis of CPNE1 mRNA expression. The hepatocellular carcinoma of the liver (LIHC) dataset from The Cancer Genome Atlas (TCGA) was selected for analysis. CPNE1 expression levels (gender, age, cancer stage, tumor grade and TP53 mutation status) in different subgroups were analyzed. Then, we analyzed the prognostic significance of CPNE1 in hepatocellular carcinoma using the Kaplan–Meier survival mapping database (http://kmplot.com) [25].

### LinkedOmics and TIMER

LinkedOmics (http://www.linkedomics.org) is a public portal containing multi-omics data from 32 cancers in TCGA [26]. In the "LinkFinder" module, we performed co-expression statistical analysis of CPNE1 using Spearman's test, and the results are displayed as volcano and heat maps. In the "LinkInterpreter" module, we performed gene ontology (GO), Kyoto Gene and Genome Encyclopedia (KEGG) pathway, kinase-target enrichment, miRNA-target enrichment and transcription factor-target enrichment analysis by gene set enrichment analysis (GSEA). The screening criteria were set as false discovery rate (FDR) < 0.05, and the number of simulations was 1000. We assessed the correlation between CPNE1 expression and immune infiltration using the Tumor Immunization Estimation Resource (TIMER) database (https://cistrome.shinyapps.io/timer/) [27]. The TISIDB (http://cis.hku.hk/TISIDB/index.php) database was used to further explore the relations between the CPNE1 expression and immune subtypes [28].

### Cell culture

Normal hepatocytes L02 as well as five human hepatoma cell lines MHCC-97H, HepG2, Hep3B, HLF and Huh7 were obtained from the Institute of Liver Diseases (Tongji Hospital, Wuhan, China) and preserved in Dulbecco's modified Eagle medium (DMEM, Hyclone), which contains 10% fetal bovine serum (FBS). Cells were incubated in an incubator containing 5% CO_2_ at 37 °C.

### Transfection

CPNE1-siRNA and overexpression plasmid were synthesized by GeneChem Co, Ltd (Shanghai, China). Suspensions of MHCC-97H, HepG2 and Hep3B cells were prepared and diluted to 6 × 10^5^/ml. Subsequently, 500 μl of cell suspensions were inoculated into 6-well plates and incubated for 24 h. The siCPNE1 or overexpression plasmid was transfected into hepatocellular carcinoma cells using Lipofectamine 3000 Transfection Reagent (Invitrogen, USA) according to the manufacturer's protocol. The sequences of CPNE1-siRNA were as follows: CPNE1-siRNA1 sequence: sense strand, 5′-GAAUCUAUGACAUAGACAATT-3′; antisense strand, 5′-UUGUCUAUGUCAUAGAUUCTT-3′, CPNE1-siRNA2 sequence: sense strand, 5′-GCAGCGUGGUUCAGGACUATT-3′; antisense strand, 5′-UAGUCCUGAACCACGCUGCTT-3′, CPNE1-siRNA3 sequence: sense strand, 5′-GCUUUGAGACAGUCCAGAATT-3′; antisense strand, 5′-UUCUGGACUGUCUCAAAGCTT-3′. The sequence of CPNE1 overexpression plasmid was showed in Additional file 6: Table S1.

### RNA extraction and real-time PCR assay

Total RNA was extracted using Trizol reagent (Invitrogen, Carlsbad, USA) and the manufacturer’s manual was followed. Complementary DNA for reverse transcription was synthesized by the Prime Script RT kit (Takara, Tokyo, Japan). Real-time PCR analysis was then performed. The 2^-ΔΔCt^ method was applied to determine differences between multiple samples. CPNE1 primer sequence: sense strand, 5′-ACCCACTCTGCGTCCTT-3′; antisense strand, 5′-TGGCGTCTTGTTGTCTATG-3′.

### Protein blotting analysis

The primary antibodies were as follows: anti-GAPDH antibody (Proteintech, 10,494–1-AP, 1:10,000), anti-CPNE1 antibody (Abcam, ab155675, 1:1000), anti-AKT antibody (CST, #4691, 1:1000), anti-phosphoAKT (CST, #4060, 1:1000) and anti-P53 antibody (CST, #2524, 1:1000). Anti-rabbit IgG (Promoter, Wuhan, China, 1:5000) and anti-mouse IgG (Promoter, Wuhan, China, 1:5000) were used as secondary antibodies.

### CCK8 and clone formation

Cell proliferation capacity was measured by Cell Counting Kit 8 (CCK-8, Promotor, Wuhan, China) according to the instructions. After adding CCK-8 reagent to 96-well plates, the cells were incubated for 2 h. The absorbance at 450 nm (OD450) was recorded. Clone formation assay was used to assess the clonogenic ability of HCC cells. Monolayers (2 × 10^3^/well) were inoculated into 6-well plates. Afterwards, cells were continuously cultured in DMEM (promoter, Wuhan, China), which was spiked with fetal bovine serum (10%, Gibco, Grand Island, NY, USA). 2 weeks later, colonies were fixed in an incubator at 37 °C for 15 min using methanol and then stained with crystal violet (0.5%, Promoter, Wuhan, China) for 15 min. The number of colonies was counted under an optical microscope.

### Transwell migration assay

Hepatocellular carcinoma cells (5 × 10^4^/ml), digested with 0.25% trypsin and conditioned with serum-free DMEM to a density of 1 × 10^5^ cells/ml, were transferred to the upper chamber. DMEM medium (600 μl) containing 10% FBS was added to the lower chamber. After incubation in a 5% CO_2_, 37 °C incubator for 24 h, the cells remaining on top of the Transwell membrane were removed with a cotton swab, and the cells migrating to the lower surface of the cells were fixed with methanol for 10 min and then stained with 0.1% crystal violet staining solution for 20 min. Images of migrating cells were taken by inverted microscopy. Five fields of view were randomly selected and the stained calls were counted. Repeat the experiment three times.

### GSEA analysis

We downloaded the RNA-seq profile data of HCC patients (project: TCGA-LUAD) from the TCGA database, and the patients were divided into high- and low-CPNE1 expression groups according the median of CPNE1 expression. Next, we performed GSEA analysis between high- and low-CPNE1 expression groups by using GSEA software (v.4.1.0), the GO: BP gene sets were used as annotated gene sets. FDR < 0.25 was considered to be significant.

### Statistical analysis

All statistical analyses were performed GraphPad Prism 8.0 software. Kaplan–Meier survival analysis and log-rank test were used to compare the survival differences between the two groups; correlation analysis was performed according to Spearman's correlation coefficient. Other experimental data were compared between the two groups by t-test, and differences were considered statistically different at P < 0.05. *P < 0.05, **P < 0.01, ***P < 0.001.

## Results

### High expression of CPNE1 in LIHC

**Fig. 1:**
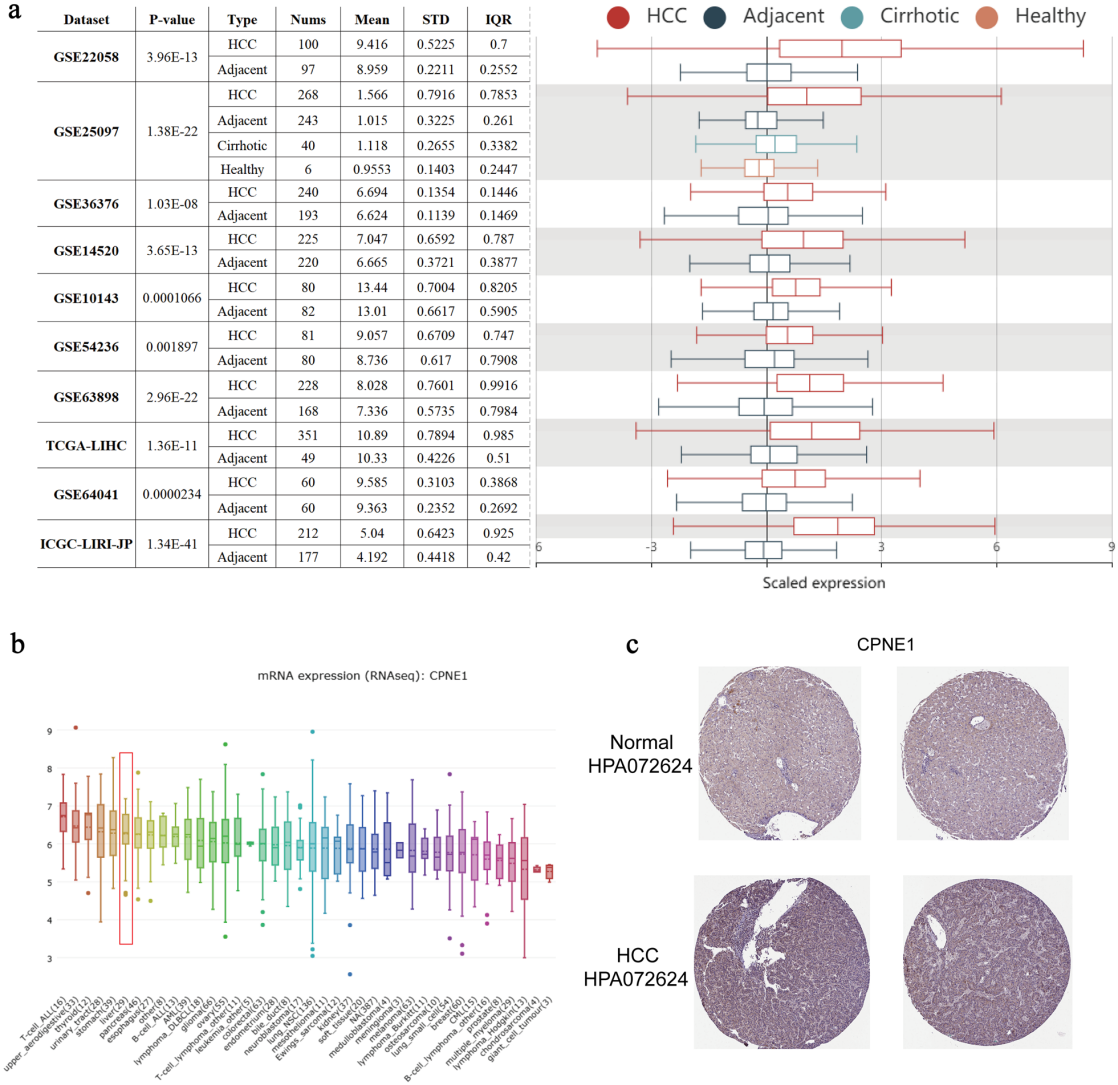
The expression of CPNE1 in LIHC. **a** Chart and plot displaying the expression of CPNE1 in cancer tissues and the adjacent normal tissues (HCCDB). **b** The mRNA expression of CPNE1 in multiple cancer cell lines (CCLE). **c** Protein expression of CPNE1 (HPA)

To evaluate the expression level of CPNE1 in HCC tissue and adjacent normal tissue, we analyzed 10 HCC cohorts in HCCDB database and found the mRNA level of CPNE1 in HCC tissue was obviously higher than in adjacent normal tissues (Fig. 1a). CPNE1 is overexpressed in HCC cell lines compared with most tumor types, which was obtained by the Cancer Cell Line Encyclopedia (CCLE) (Fig. 1b). Moreover, we used HPA database to explore the protein expression of CPNE1. Compared to normal liver tissue, HCC tissue exhibited CPNE1 strong positive staining. Here, we presented the representative images of immunohistochemistry for HCC tissues and normal liver tissues (Fig. 1c). All results suggested the expression level of CPNE1 was significantly upregulated in HCC.

**Fig. 2:**
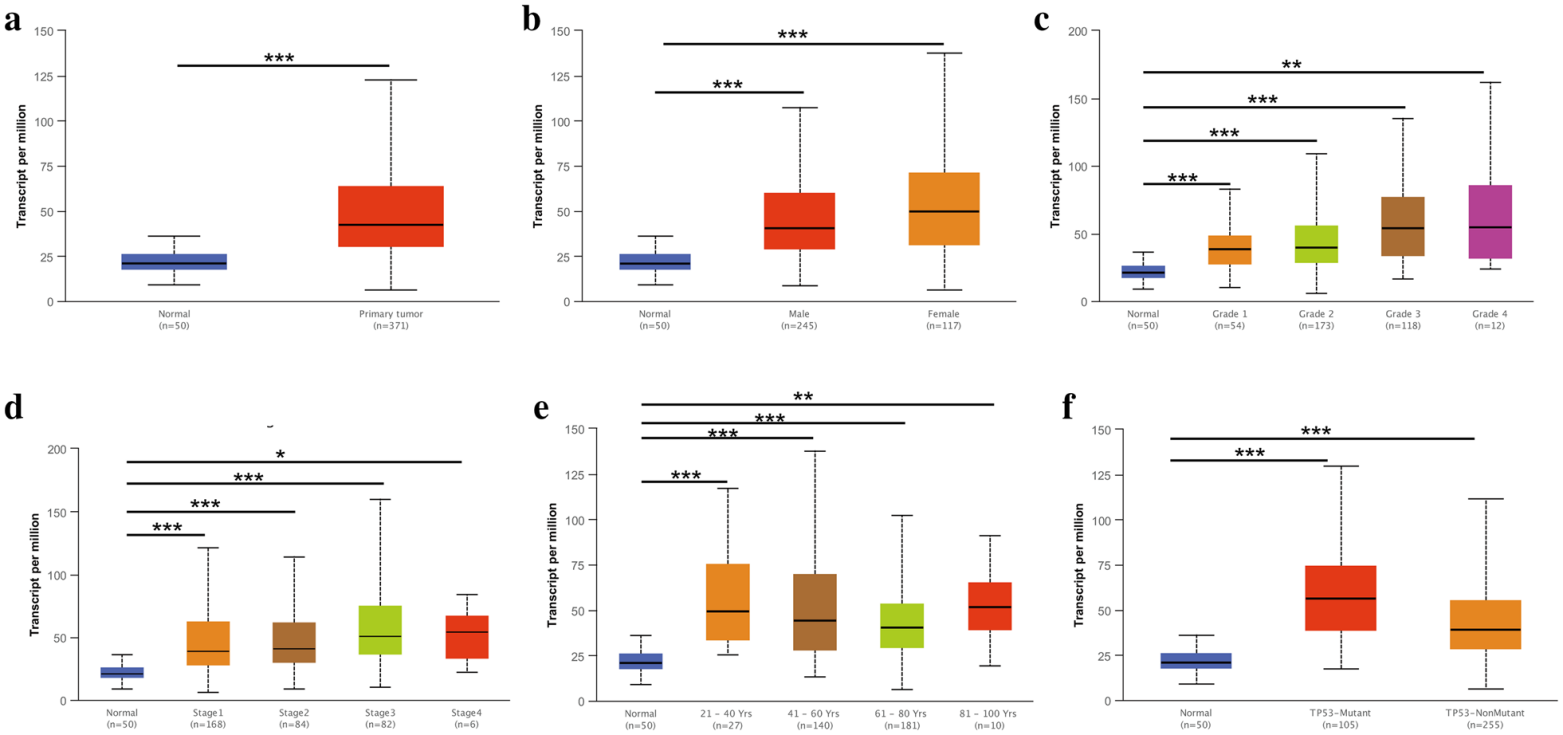
Subgroup expression analysis of CPNE1 in LIHC (UALCAN). **a** The RNA expression of CPNE1 in normal and LIHC patients. **b** The mRNA expression of CPNE1 in normal and LIHC patients with different genders. **c** The mRNA expression of CPNE1 in normal and LIHC patients with different tumor grades. **d** The mRNA expression of CPNE1 in normal and LIHC patients with different tumor stages. **e** The mRNA expression of CPNE1 in normal and LIHC patients with different ages. **f** The mRNA expression of CPNE1 in normal and LIHC patients with different TP‐53 mutant status

To enhance the credibility of the above results, we evaluated the high expression of CPNE1 in LIHC sample from TCGA through the UALCAN database. Compared with the normal samples (n = 50), the mRNA level of CPNE1 was higher in the LIHC samples (n = 371) (Fig. 2a). By Subgroup analysis, we found that CPNE1 was also highly expressed in the subgroups of sex and age (Fig. 2b, e). In terms of tumour stage and cancer grade, we found CPNE1 was highly expressed in grades 1‐4 and stages 1‐4 (Fig. 2c, d). Furthermore, CPNE1 was evidently linked to TP53 mutation and was markedly upregulated in LIHC patients with TP53 mutations (Fig. 2f). Collectively, these data implicated that the overexpression of CPNE1 was strongly linked to LIHC progression.

### The prognostic value of CPNE1 in LIHC patients

**Fig. 3:**
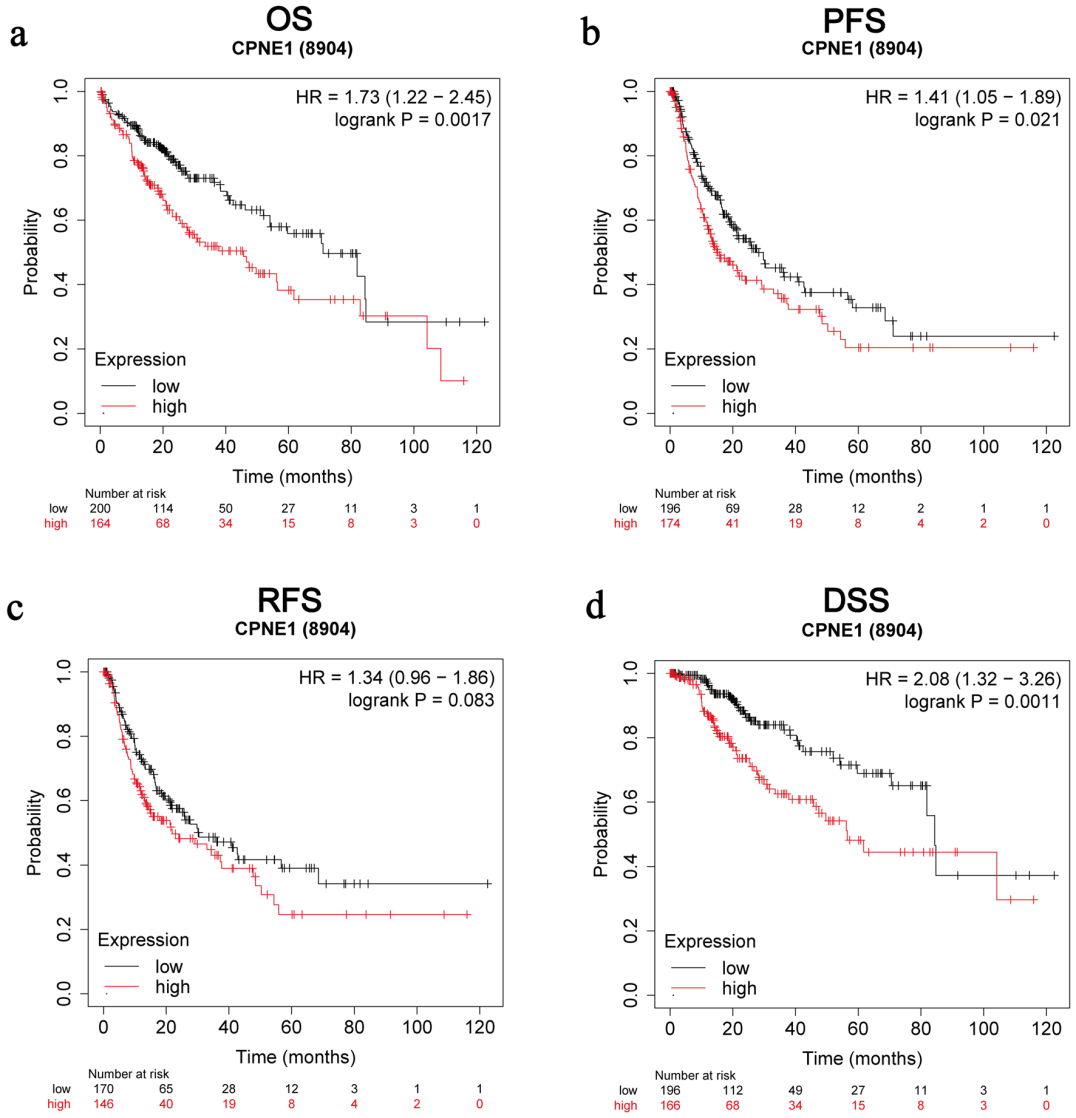
High expression of CPNE1 predicts poor prognosis in LIHC (Kaplan‐Meier Plotter). **a** The prognostic values of CPNE1 in OS of LIHC patients. **b** The prognostic values of CPNE1 in PFS of LIHC patients. **c** The prognostic values of CPNE1 in RFS of LIHC patients. **d** The prognostic values of CPNE1 in DSS of LIHC patients

By using Kaplan‐Meier Plotter database, we explored the prognostic valences of CPNE1 in LIHC patients (n = 364). The overexpression of CPNE1 was evidently linked to poor overall survival (OS, HR = 1.73, log-rank P = 0.0017), progression‐free survival (PFS, HR = 1.41, log-rank P = 0.021), relapse‐free survival (RFS, HR = 1.34, log-rank P = 0.083) and disease‐specific survival (DSS, HR = 2.08, log-rank P = 0.0011) of LIHC patients (Fig. 3a–d). In all, the overexpression of CPNE1 was linked with poor prognosis of LIHC patients.

Using the Kaplan–Meier Plotter database, we investigated the correlations between CPNE1 expression and clinicopathologic features of LIHC patients to better find out the effect of CPNE1 on the survival of LIHC patients (Tables 1, 2). CPNE1 was linked with poor OS in many patients with LIHC, other than those with Female (HR = 1.7, P = 0.063), stage 1 (HR = 1.66, P = 0.15), or grade 3 (HR = 1.64, P = 0.11), or hepatitis (HR = 1.28, P = 0.46) (Table 1). In terms of RFS, compared to grade 2/3, the HR values for RFS in patients with grade 1 indicated significant statistic differences (Table 1). In terms of PFS, CPNE1 had no significance in stage 1 (HR = 1.32, P = 0.3) or stage 2 (HR = 0.53, P = 0.052) patients, those with grade 3 (HR = 0.72, P = 0.21), hepatitis patients (HR = 0.63, P = 0.066), or those with alcohol consumption (HR = 0.62, P = 0.099) (Table 2). In terms of DSS, compared to stage 2/3, the HR values for DSS in patients with stage 1 indicated no statistic difference (Table 2). Furthermore, compared to hepatitis patients, the HR values for PFS and OS in patients without hepatitis indicated significant statistic differences (Table 1). These results showed that overexpression of CPNE1 may decline survival in patients without hepatitis.

**Table 1:** Correlation between CPNE1 mRNA expression and OS and RFS in HCC based on different clinical parameters by Kaplan–Meier plotter

Clinical characteristics	OS (N = 364)			RFS (N = 316)		
	N	HR	*P*-value	N	HR	*P*-value
Gender						
Female	118	1.7 (0.97–3)	0.063	106	2.02 (1.11–3.66)	**0.019**
Male	246	1.74 (1.12–2.71)	**0.013**	210	0.66 (0.43–1.02)	0.061
Stage						
I	170	1.66 (0.83–3.32)	0.15	153	0.81 (0.47–1.41)	0.46
II	83	2.24 (1–5.03)	**0.045**	75	0.48 (0.23–0.98)	**0.039**
III	83	2.53 (1.37–4.86)	**0.0022**	70	2.04 (1.08–3.85)	**0.026**
IV	4	NA	NA	0	NA	NA
Grade						
1	55	3.16 (1.19–8.41)	**0.015**	45	4.1 (1.48–11.38)	**0.0036**
2	174	1.77 (1.06–2.96)	**0.027**	149	1.62 (0.99–2.65)	0.055
3	118	1.64 (0.89–3.05)	0.11	107	0.67 (0.39–1.17)	0.16
4	12	NA	NA	11	NA	NA
Hepatitis virus						
Yes	150	1.28 (0.67–2.45)	0.46	139	0.6 (0.35–1.01)	0.053
None	167	1.85 (1.15–2.96)	**0.0097**	143	2.22 (1.33–3.71)	**0.0017**
Alcohol consumption						
Yes	115	2.3 (1.18–4.49)	**0.012**	99	0.65 (0.33–1.29)	0.22
None	202	1.83 (1–3.34)	**0.046**	183	1.56 (1–2.45)	0.05

*OS* Overall Survival, *RFS* Relapse Free Survival, *HR* Hazard ratio

**Table 2:** Correlation between CPNE1 mRNA expression and PFS and DSS in HCC based on different clinical parameters by Kaplan–Meier plotter

Clinical characteristics	PFS (N = 370)			DSS (N = 362)		
	N	HR	*P*-value	N	HR	*P*-value
Gender						
Female	121	2.11 (1.26–3.53)	**0.038**	118	2.06 (0.98–4.33)	0.051
Male	249	0.64 (0.43–0.94)	**0.023**	244	2.06 (1.16–3.64)	**0.011**
Stage						
I	171	1.32 (0.78–2.25)	0.3	168	1.71 (0.69–4.22)	0.24
II	85	0.53 (0.28–1.02)	0.052	83	3.37 (1.13–10.06)	**0.021**
III	85	2.15 (1.2–3.82)	**0.0082**	83	2.97 (1.41–6.26)	**0.0029**
IV	5	NA	NA	3	NA	NA
Grade						
1	55	3.83 (1.67–8.75)	**0.00069**	55	3.24 (0.91–11.53)	0.056
2	177	1.65 (1.05–2. 6)	**0.03**	171	2.31 (1.17–4.54)	**0.013**
3	121	0.72 (0.43–1.21)	0.21	119	2.07 (0.93–4.61)	0.068
4	12	NA	NA	12	NA	NA
Hepatitis virus						
Yes	153	0.63 (0.39–1.03)	0.066	151	1.85 (0.81–4.23)	0.14
None	169	2.4 (1.54–3.75)	**7.7e−05**	165	2.52 (1.37–4.65)	**0.0022**
Alcohol consumption						
Yes	117	0.62 (0.35–1.1)	0.099	117	2.5 (1.16–5.41)	**0.016**
None	215	1.68 (1.13–2.52)	**0.01**	199	2.9 (1.48–5.7)	**0.0012**

*PFS* Progression Free Survival, *DSS* Disease Specific Survival, *HR* hazard ratio

### Co-expression genes of CPNE1 and enrichment analysis in patients with LIHC

**Fig. 4:**
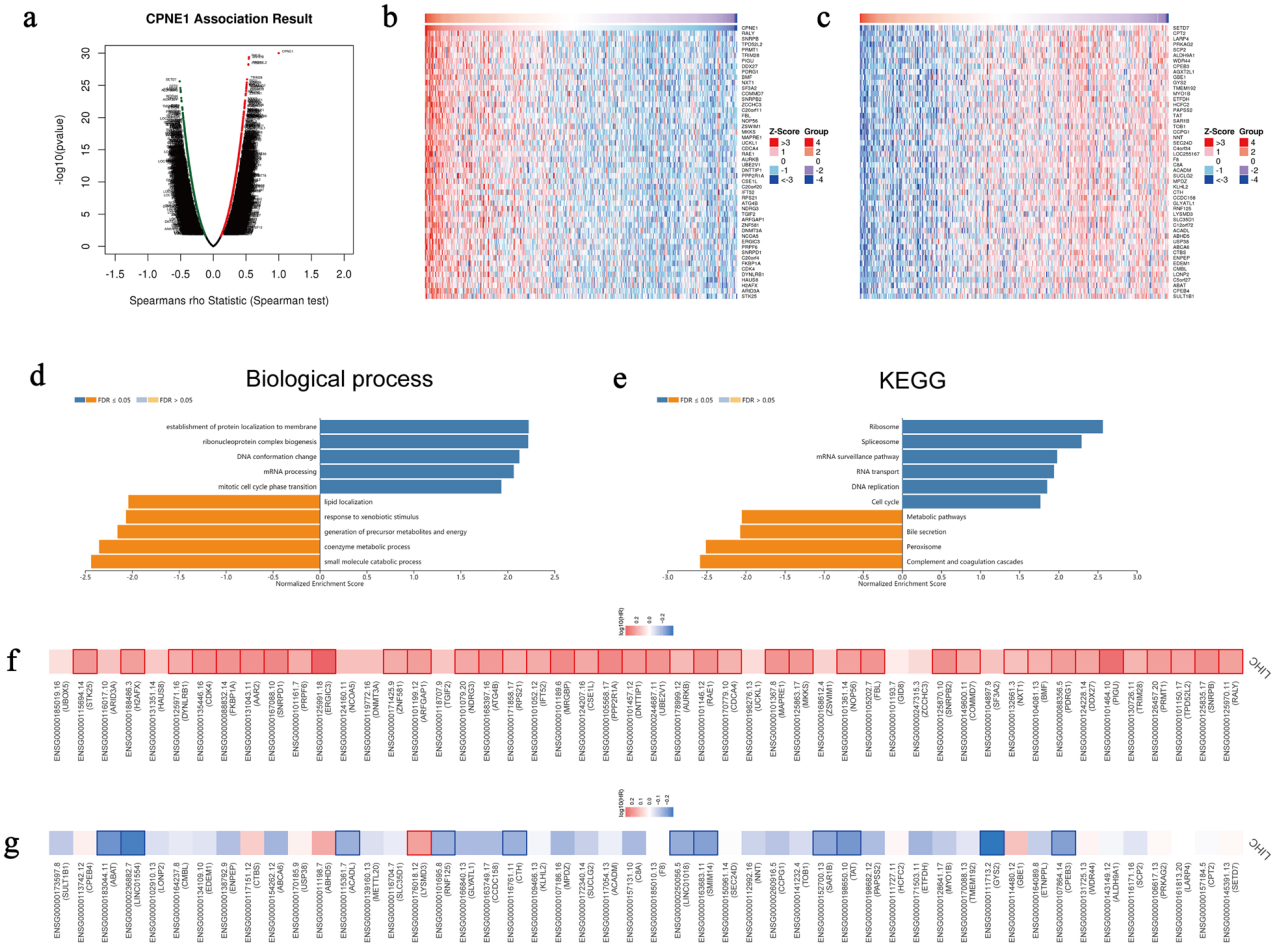
Co-expression genes of CPNE1 in LIHC (LinkedOmics). **a** Correlations between CPNE1 and differently expressed genes (Spearman test). **b**, **c** Heat maps showing top 50 genes positively and negatively correlated with CPNE1 in LIHC. **d** Enrichment GO: Biological process annotations analysis of CPNE1 in LIHC. **e** Enrichment KEGG pathways of CPNE1 in LIHC. **f** Survival map of the top 50 genes positively correlated with CPNE1 in LIHC. **g** Survival map of the top 50 genes negatively correlated with CPNE1 in LIHC

To further elucidate the importance of CPNE1 in LIHC, we explored coexpression patterns of CPNE1 using LinkFinder module in LinkedOmics. The result showed 5,896 genes (dark red dots) were related positively to CPNE1, while 3,780 genes (dark green dots) were related negatively to CPNE1 in LIHC (FDR < 0.05) (Fig. 4a). Additionally, the top 50 genes clearly related (positively and negatively) to CPNE1 were displayed in Fig. 4b and Fig. 4c. CPNE1 expression showed an obvious positive link with expression of RALY (r = 0.545, FDR = 4.06E−26), SNRPB (r = 0.543, FDR = 4.86E−26), TPD52L2 (r = 0.536, FDR = 2.51E−25) and PRMT1 (r = 0.536, FDR = 2.51E−25). Notably, the top 50 clearly positive genes demonstrated the high possibility of being high risk genes in LIHC, in which 39/50 genes presented high HR (hazard ratio) (P < 0.05) **(**Fig. 4f). By contrast, among the top 50 negatively correlated genes, there were 12/50 genes with low HR (P < 0.05) (Fig. 4g).

Subsequently, we conducted Functional Enrichment Analysis. GO term revealed that CPNE1 and its coexpressed genes were primarily involved in the establishment of protein localization to membrane, ribonucleoprotein complex biogenesis, lipid localization and response to xenobiotic stimulus (Fig. 4d). KEGG results showed these genes were mainly enriched in ribosome, spliceosome, metabolic pathways and bile secretion (Fig. 4e).

### Regulators of CPNE1 networks in LIHC

To further explore the possible regulators of CPNE1 in LIHC, we analysed networks of transcription factor (TF), miRNA or kinase targets of CPNE1 co-expression genes. Kinases ATR, CHEK1, PLK3, CHEK2 and DAPK1 were the top 5 most important targets. Interestingly, CPNE1 co-expression genes were not enriched in any significant miRNA targets. TF enrichment results revealed CPNE1 co-expression genes were predominantly enriched in E2F transcription factor family, including V$E2F1DP2_01, V$E2F_02, V$E2F1_Q6_01, V$E2F1DP1_01 and V$E2F1DP2_01 (Table 3). Results above suggested that CPNE1 had wide-ranging impact on overall transcriptome in LIHC.

**Table 3:** The Kinases, miRNA and transcription factors-target networks of CPNE1 in LIHC (LinkedOmics)

Enriched Category	Geneset	LeadingEdgeNum	FDR
Kinase Target	Kinase_ ATR	29	0
	Kinase_ CHEK1	46	0
	Kinase_ PLK3	12	0.0011024
	Kinase_ CHEK2	11	0.0014699
	Kinase_ DAPK1	8	0.0022048
miRNA Target	ATGTACA,MIR-493	70	0
	CTTGTAT,MIR-381	65	0
	TGCACGA,MIR-517A,MIR-517C	3	0.041935
	CACGTTT,MIR-302A	6	0.048338
	AGTCTTA,MIR-499	19	0.060606
Transcription Factor	SGCGSSAAA _V$E2F1DP2_01	63	0.0016107
	V$E2F_02	79	0.0020939
	V$E2F1_Q6_01	79	0.0021476
	V$E2F1DP1_01	79	0.0023265
	V$E2F1DP2_01	79	0.0023265

### The expression level of CPNE1 in LIHC cell lines and the construction of knockdown cell lines

**Fig. 5:**
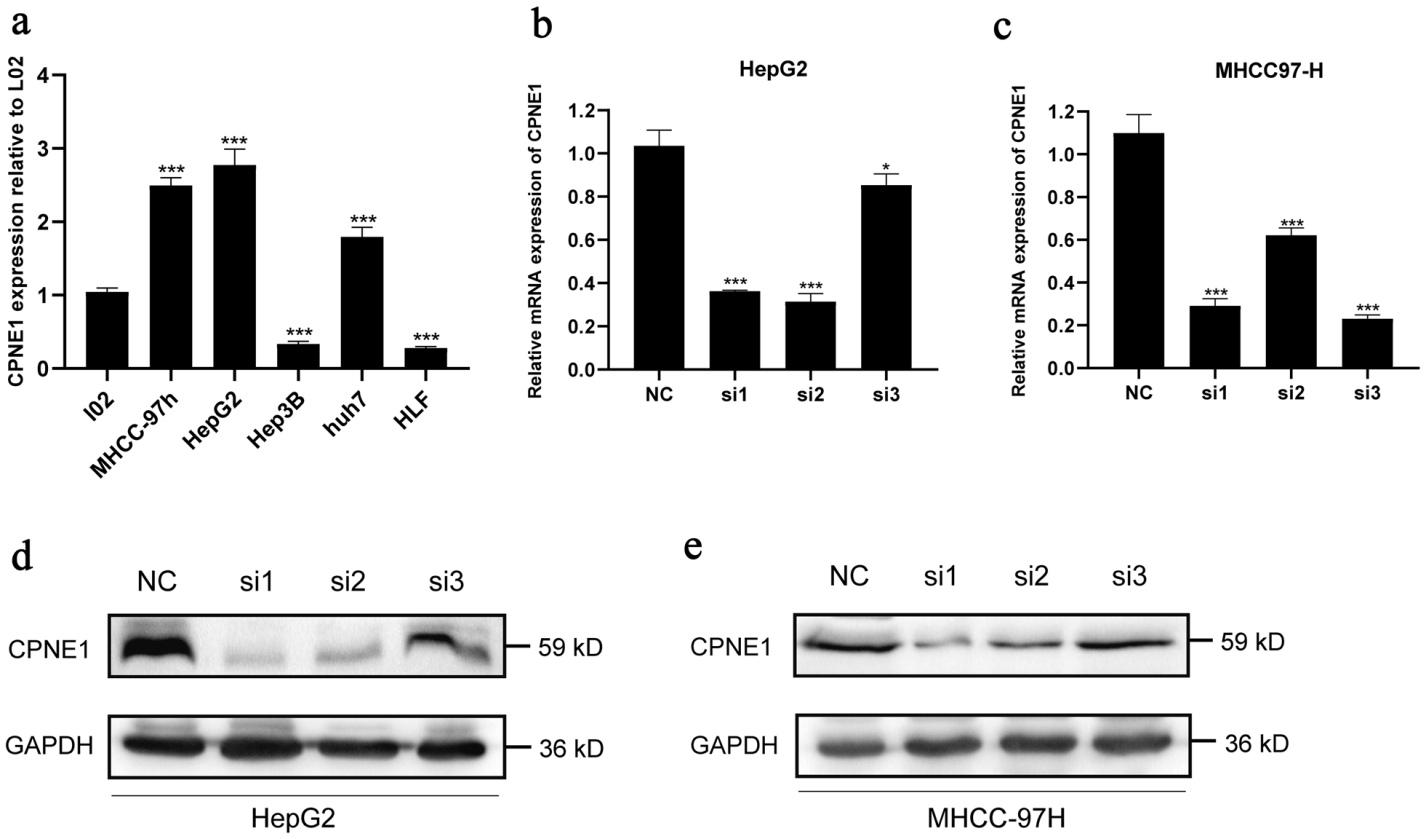
The expression of CPNE1 in LIHC cell lines and the construction of CPNE1 knockdown cell lines. **a** The mRNA expression level of CPNE1 in LIHC cell lines by RT-qPCR. **b** The mRNA expression level of CPNE1 in HepG2 transfected CPNE1-siRNA by RT-qPCR. **c** The mRNA expression level of CPNE1 in MHCC-97H transfected CPNE1-siRNA by RT-qPCR. **d** The protein expression level of CPNE1 in HepG2 transfected CPNE1-siRNA by Western Blot. **e** The protein expression level of CPNE1 in MHCC-97H transfected CPNE1-siRNA by Western Blot

To validate whether CPNE1 was overexpressed in LIHC cell lines, we tested the mRNA expression level of CPNE1 in L02 (a human normal liver cell line) and 5 human hepatoma cell lines (MHCC-97H, HepG2, Hep3B, Huh7 and HLF) by using RT-qPCR. Compared with L02 cell, the expression level of CPNE1 in HepG2, MHCC-97H and huh7 was much higher, which indicated the expression level of CPNE1 in human hepatoma cell lines was higher than that in human normal liver cell line (Fig. 5a). The result was consistent with our bioinformatic analysis.

From the results above, we selected HepG2 and MHCC-97H for the subsequent experiments and constructed CPNE1 knockdown cell lines. We chose 3 RNA interference targets (CPNE1-si1, CPNE1-si2, CPNE1-si3) transiently transfected into HepG2 and MHCC-97H cells. The knockdown efficiency of CPNE1 was detected by Western Blot and RT-qPCR. Compared with the negative control group, the expression level of CPNE1 in CPNE1-siRNA transfected cells was significantly decreased (Fig. 5b–e, Additional file 1: Figure S1). Of these, CPNE1-si1 and CPNE1-si2 revealed a higher knockdown efficiency in HepG2 and MHCC-97H cells so we selected CPNE1-si1 and CPNE1-si2 for the subsequent experiments.

### Effects of CPNE1 on LIHC cell proliferation

**Fig. 6:**
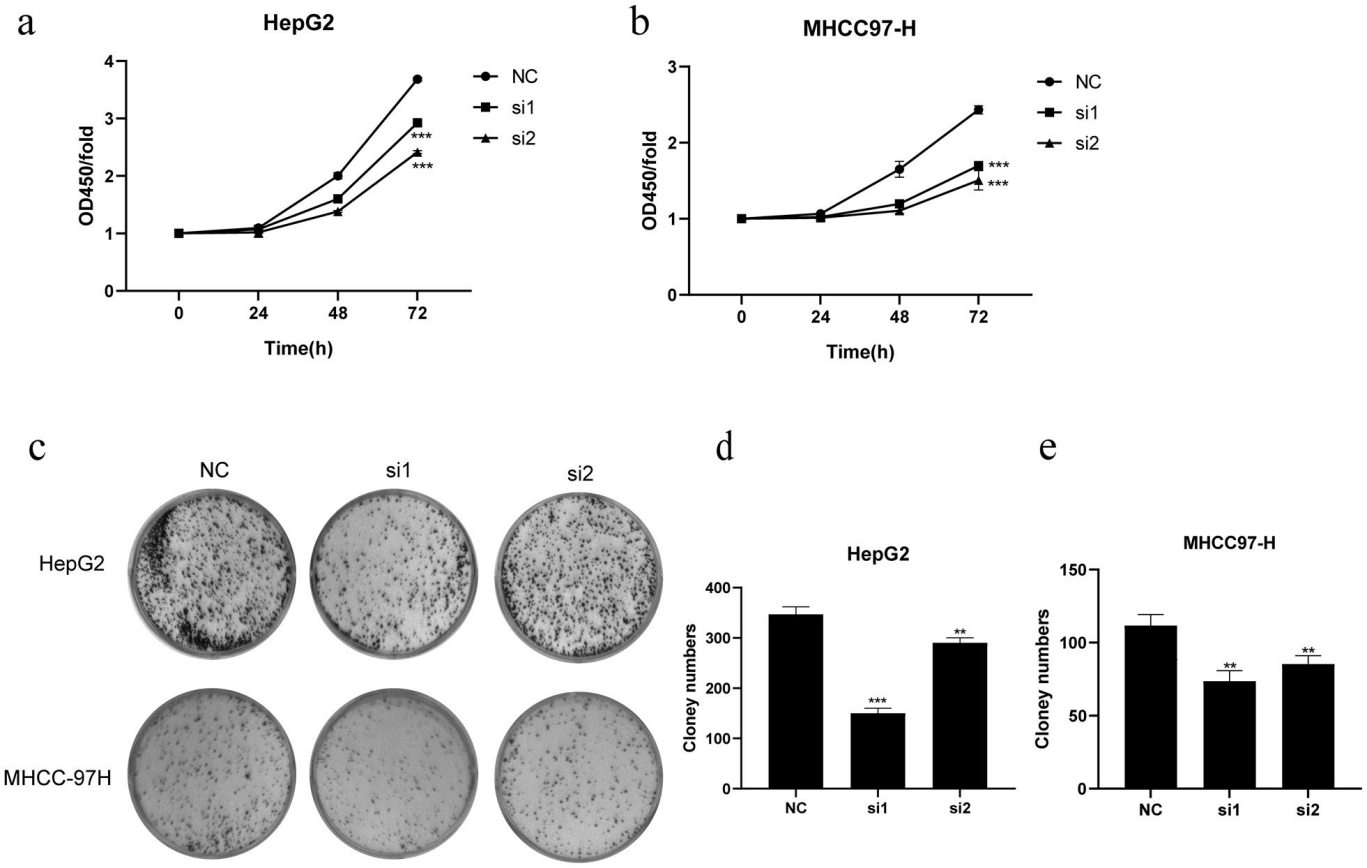
Effects of CPNE1 on cell proliferation in LIHC. **a** Effects of CPNE1 on cell viability in HepG2 by CCK8. **b** Effects of CPNE1 on cell viability in MHCC97-H by CCK8. **c**–**e** Effects of CPNE1 on cell clonal formation ability in HepG2 and MHCC97-H by plate clone formation assay

CCK8 assay was performed to test the difference of cell viability between negative control group and CPNE1-siRNA transfected group. The result showed the OD value of CPNE1-siRNA transfected cells was much smaller than control group after 48 h, which indicated the cell viability of CPNE1 knockdown cell lines was considerably reduced in HepG2 and MHCC-97H cells (Fig. 6a, b). In addition, plate clone formation assay revealed the clone numbers of CPNE1-siRNA transfected cells were less than control group in HepG2 and MHCC-97H cells (Fig. 6c–e). Above results showed that knockdown of CPNE1 inhibited LIHC cells proliferation.

### Effects of CPNE1 on LIHC cell migration

**Fig. 7:**
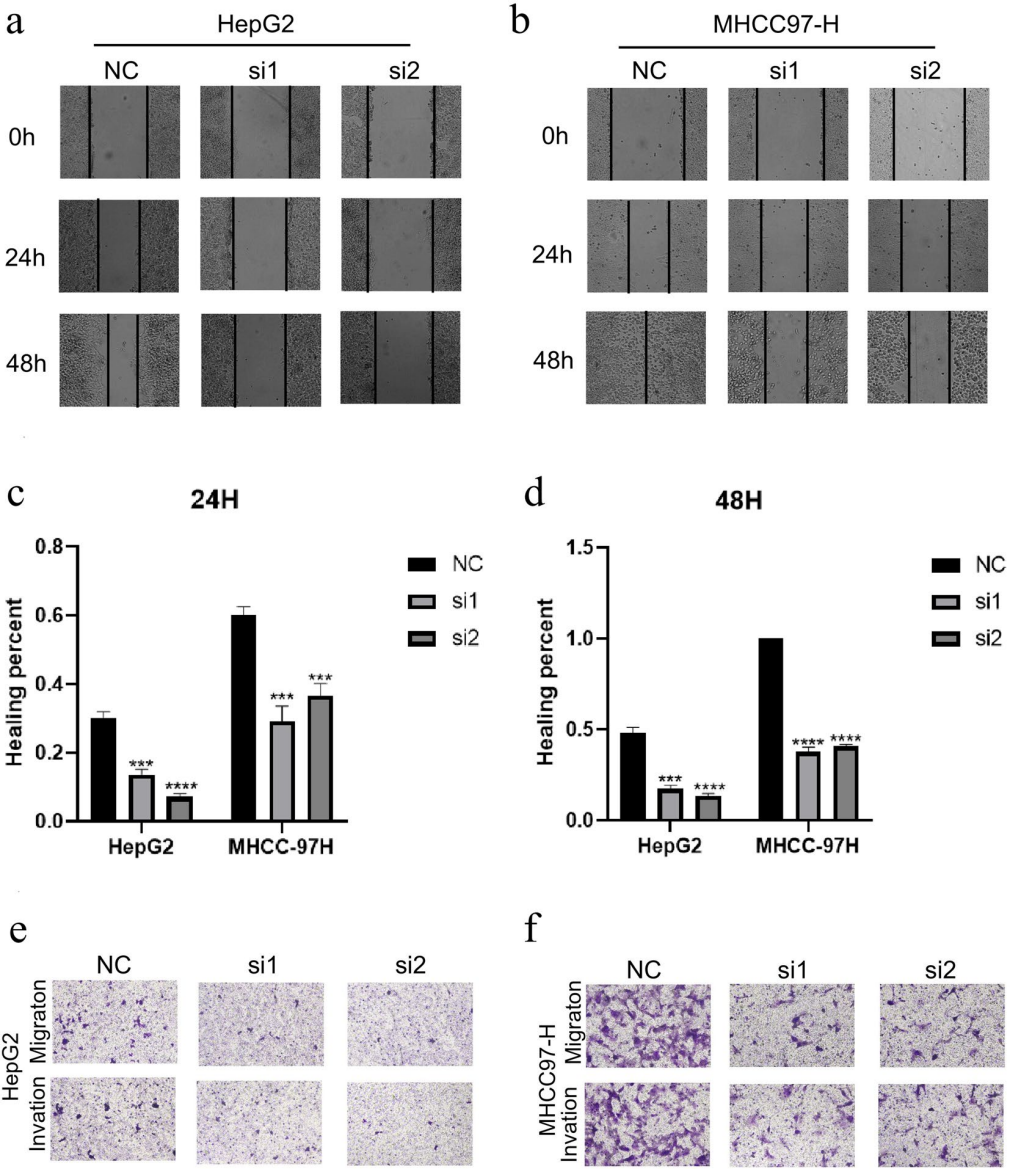
Effects of CPNE1 knockdown on cell migration in LIHC. **a**–**d** Effects of CPNE1 on cell migration by using scratch assay in HepG2 and MHCC-97H cells. **e**, **f** Effects of CPNE1 on cell migration by using Transwell assay in HepG2 and MHCC-97H cells

To further explore the correlations between CPNE1 and LIHC cell migration, the scratch assay was performed to test the impact of CPNE1 on LIHC cell migration. The result showed the healing rate of low CPNE1 expression group significantly reduced in comparison to control group in HepG2 and MHCC-97H cells (Fig. 7a–d). This suggested that CPNE1 was related with the migration ability of HepG2 and MHCC-97H cells. Meanwhile, Transwell assay was conducted to validate the effect of CPNE1 on LIHC cell lines migration and invasion capabilities. Consistent with the scratch assay results, in HepG2 and MHCC-97H cells, the number of CPNE1 knockdown cells traversing to the lower chamber was less than that in control group (Fig. 7e, f). These results indicated that CPNE1 participated in the regulation of migration and invasion capabilities in LIHC cell lines.

### Effects of CPNE1 knockdown on AKT/P53 pathway in LIHC

**Fig. 8:**
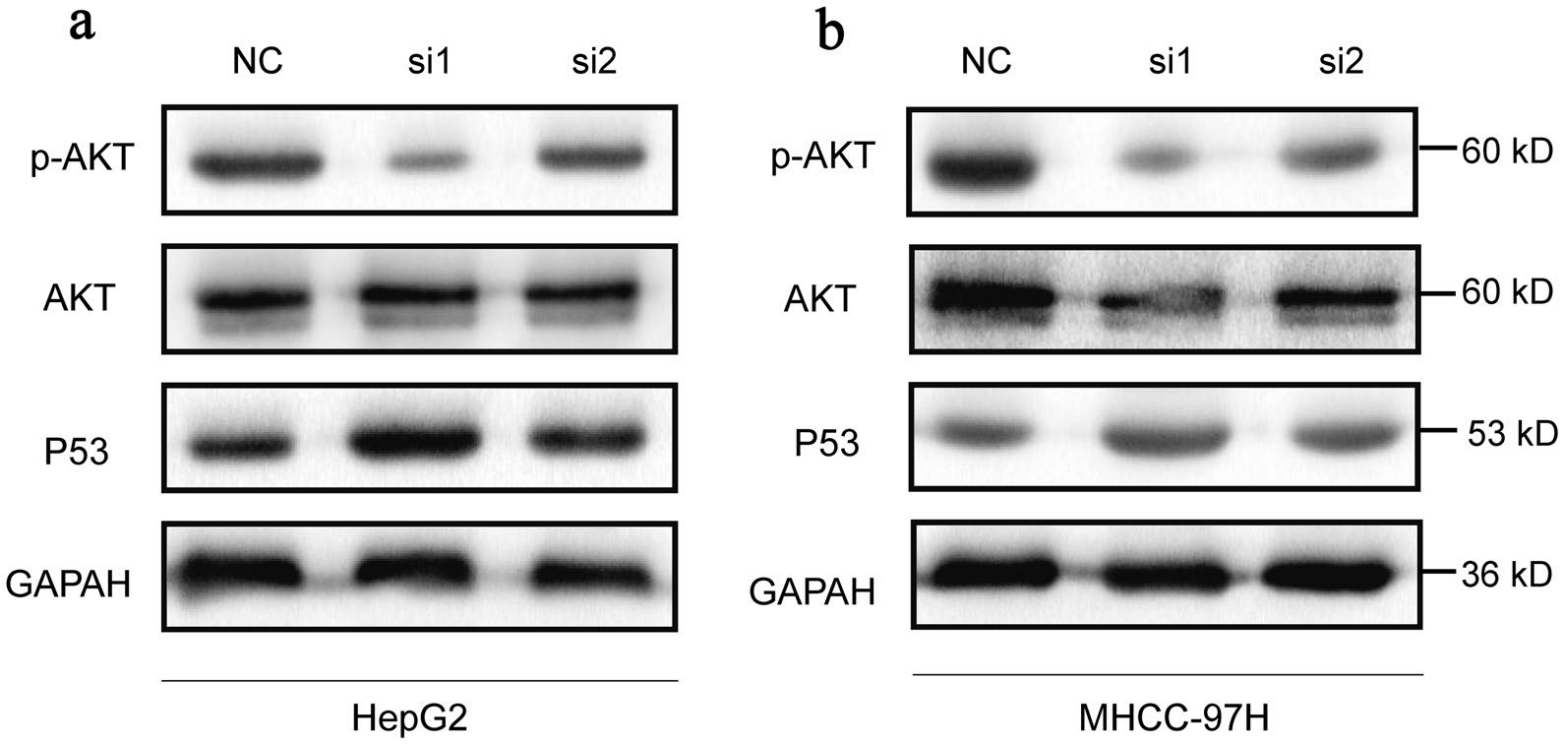
Effects of CPNE1 knockdown on AKT/P53 pathway in LIHC. **a** Effects of CPNE1 knockdown on AKT/P53 pathway in HepG2 cell. **b** Effects of CPNE1 knockdown on AKT/P53 pathway in MHCC-97H cell

GSEA analysis revealed that AKT pathway was enriched in high-CPNE1 expression group (Additional file 2: Figure S2). Western blot experiment was further conducted to detect protein expression levels of p-AKT and P53 for further exploration in LIHC. Compared to the control group, the protein expression level of p-AKT in CPNE1-siRNA group was markedly decreased and the expression level of P53 was upregulated, while the expression level of total AKT indicated no significant change in HepG2 and MHCC-97H cells (Fig. 8a, b and Additional file 3: Figure S3). These results showed that the CPNE1 may regulate AKT/P53 pathway, thus promoting the malignant progression of HCC.

### Effects of CPNE1 overexpression on LIHC cell

**Fig. 9:**
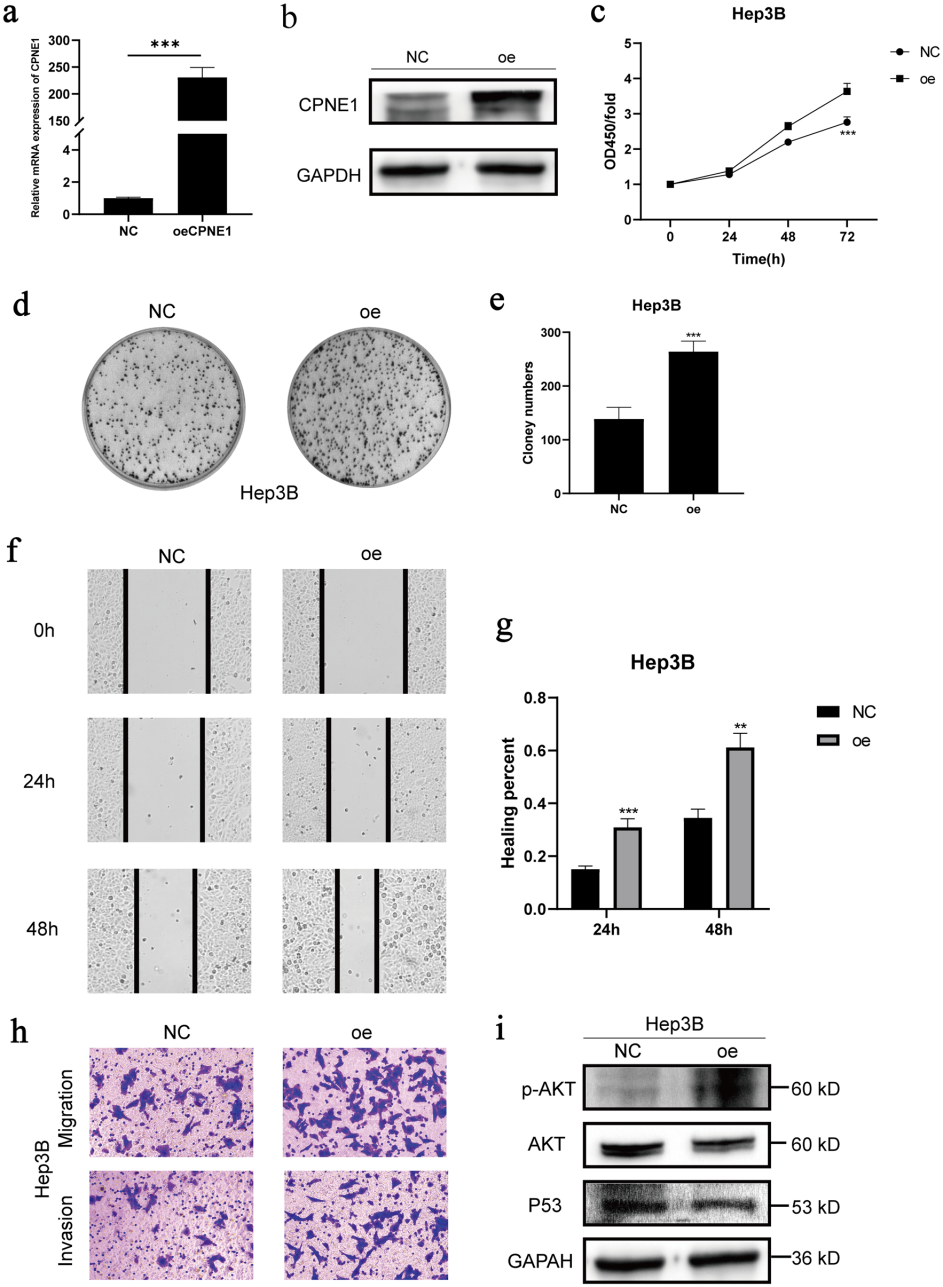
Effects of CPNE1 overexpression on cell proliferation, migration and pathway in Hep3B. **a**, **b** The expression level of CPNE1 in Hep3B transfected overexpression plasmid by PCR and Western Blot. **c**–**e** Effects of CPNE1 overexpression on cell proliferation in Hep3B by CCK8 and plate clone formation assay. **f**, **g** Effects of CPNE1 overexpression on cell migration by using scratch assay in Hep3B cell. **H **Effects of CPNE1 overexpression on cell migration by using Transwell assay in Hep3B cell. **i** Effects of CPNE1 overexpression on AKT/P53 pathway in Hep3B cell

To further study the function of CPNE1 in LIHC cell, we selected Hep3B for the subsequent experiments and constructed CPNE1 overexpression cell line. We chose plasmid transiently transfected into Hep3B cell. The transfection efficiency of CPNE1 was detected by RT-qPCR and Western Blot. Compared to the negative control group, the mRNA and protein expression level of CPNE1 in transfected cells were significantly increased (Fig. 9a, b and Additional file 4: Figure S4). We performed CCK8 assay to test the difference of cell viability between negative control group and CPNE1 overexpression group. The result indicated the OD value of CPNE1 overexpression cell was higher than control group especially after 48 h, which showed the cell viability of CPNE1 overexpression cell was considerably increased in Hep3B cell (Fig. 9c). In addition, plate clone formation assay revealed the clone numbers of CPNE1 overexpression cell were more than control group in Hep3B cell (Fig. 9d, e).

Besides, the scratch assay was performed to test the overexpression of CPNE1 on Hep3B cell migration. The result showed the healing rate of high CPNE1 expression group significantly increased in comparison to control group in Hep3B cell (Fig. 9f, g). Meanwhile, Transwell assay was conducted to validate the effect of CPNE1 on Hep3B cell line migration and invasion capabilities. Consistent with the scratch assay results, in Hep3B cell, the number of CPNE1 overexpression cells traversing to the lower chamber was more than that in control group (Fig. 9h).

Furthermore, compared to the control group, the protein expression level of p-AKT in CPNE1 overexpression group was markedly increased and the expression level of P53 was decreased, while the expression level of total AKT indicated no significant change in Hep3B cell (Fig. 9i and Additional file 5: Figure S5).

Previous studies have demonstrated that the AKT signaling pathway was activated in HCC and associated with multiple malignant biological behaviors of HCC, inhibition of AKT signaling inhibits the proliferation and migration of HCC [29, 30]. In addition, P53 act as a tumor suppressor gene, lots of studies have shown that promoting P53 signaling could suppress the progression of hepatocellular carcinoma [31, 32]. Above all, these above results illustrated that CPNE1 mediated HCC progression at least partly via regulating AKT/P53 signaling.

### Correlation of CPNE1 expression with immune infiltration and immune markers in LIHC

**Fig. 10:**
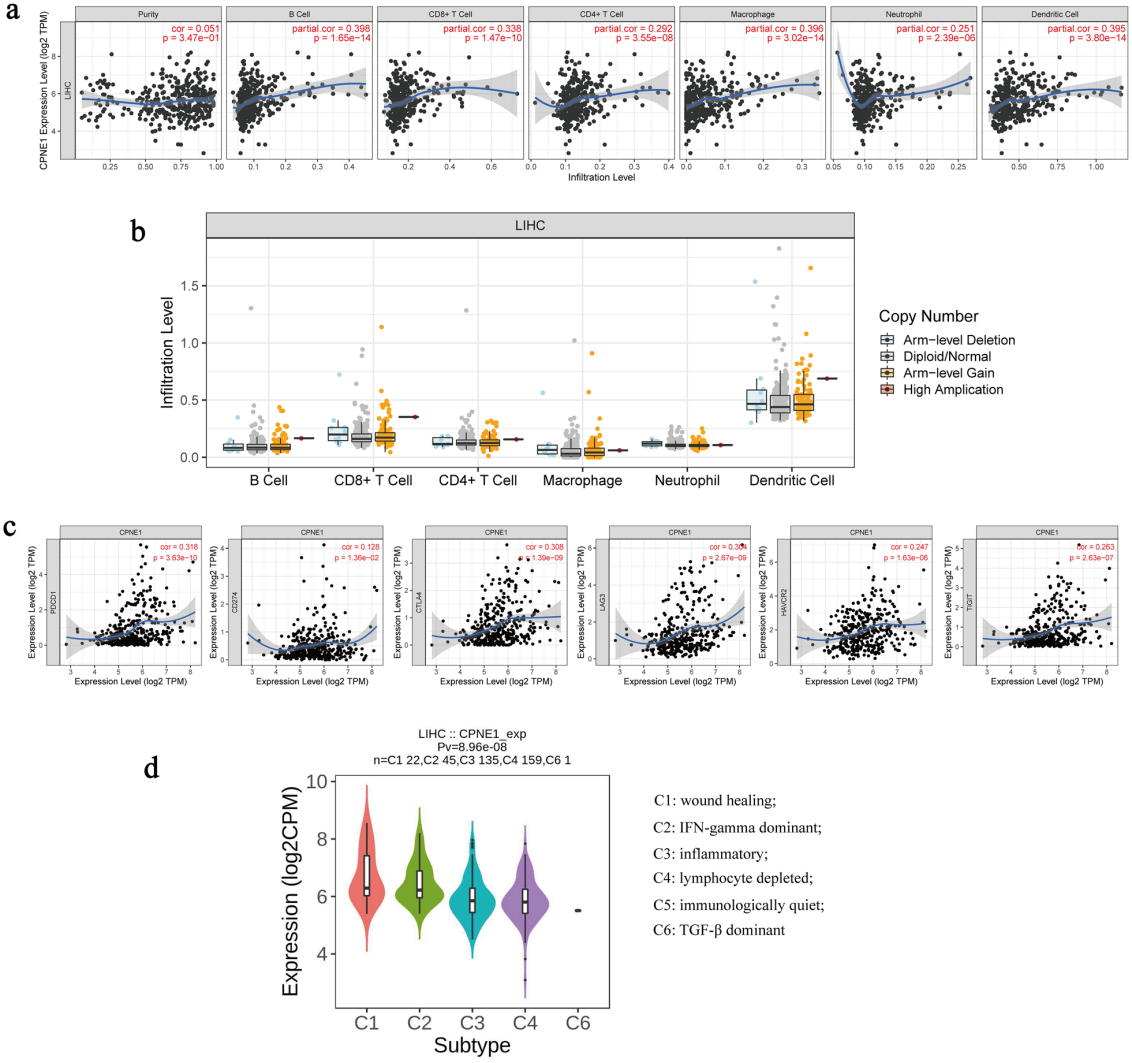
Correlations of CPNE1 expression with immune infiltration in LIHC. **a** Correlations of CPNE1 with infiltrating levels of dendritic cells, macrophages, CD8 + T cells, neutrophils, B cells and CD4 + T cells in LIHC. **b** Correlations of CPNE1 mutation with immune infiltration. **c** Correlations of CPNE1 with the expression level of multiple immune checkpoint molecules. **d** Correlation of CPNE1 expression and immune subtypes

**Fig. 11:**
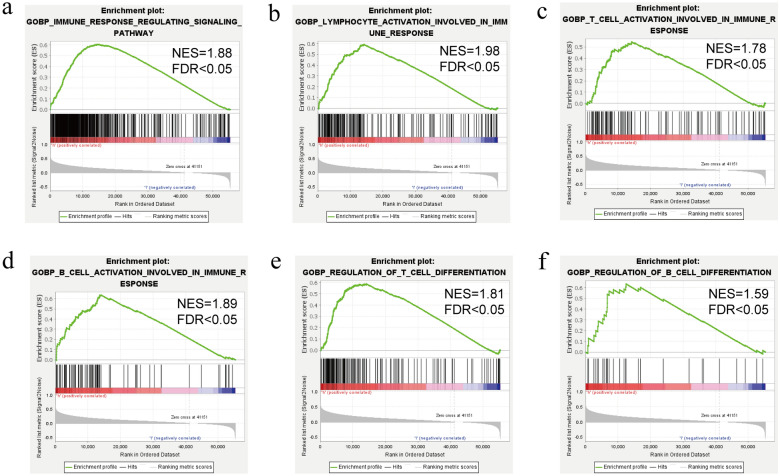
GSEA analysis between high- and low-risk groups. **a**–**f** Some lymphocyte activation involved in immune response was gathered in high-risk group: “T cell activation,” “B cell activation,” “regulation of T cell differentiation” and “regulation of B cell differentiation.” NES, Normalized enrichment score; FDR, false discovery rate

We explored the relationship of CPNE1 expression and immune infiltration using TIMER. The correlation coefficients between CPNE1 expression and the abundances of multiple immune cells (dendritic cells, neutrophils, CD8+ T cells, macrophages, CD4+ T cells and B cells) were explored using Spearman tests. We found that CPNE1 expression had no correlation with tumor purity (cor = 0.051, P = 3.47E−01). Furthermore, CPNE1 expression had significant association with all six immune cells infiltration, especially B cells (cor = 0.398, P = 1.65E−14), macrophages (cor = 0.396, P = 3.02E−14) and dendritic cells (cor = 0.395, P = 3.80E−14) (Fig. 10a). CPNE1 mutation did not impact immune infiltration (Fig. 10b). Additionally, by using Spearman correlation analysis in TIMER database, we assessed the correlation between CPNE1 expression and six immune checkpoint molecules (PDCD1, CD274, CTLA4, LAG3, HAVCR2, TIGIT) and found CPNE1 expression was strikingly positively correlated with the six immune checkpoint molecules (Fig. 10c). Then, we analyzed the relationship between CPNE1 expression and immune subtypes, which indicated that CPNE1 expression was significantly correlated to immune subtypes in LIHC (P < 0.001) (Fig. 10d). Moreover, after adjustments for tumor purity, the CPNE1 expression level was significantly correlated with 53 out of 61 immune cell markers in LIHC (Table 4). GSEA results also showed that CPNE1 was involved in some immune response regulating signaling pathways, such as lymphocyte activation involved in immune response, T cell activation involved in immune response, B cell activation involved in immune response, regulation of T cell differentiation and regulation of B cell differentiation (NES > 1*,* FDR < 0.05) in LIHC (Fig. 11a–f). All together, these results suggest that CPNE1 is critically engaged in immune infiltration during the advancement of LIHC.

**Table 4:** The relationships between CPNE1 expression and immune markers in LIHC (TIMER)

Description	Gene markers	LIHC			
		None		Purity	
		Cor	p	Cor	p
CD8 + T cell	CD8A	0.224	1.37E−05	0.304	7.86E−09
	CD8B	0.228	8.78E−06	0.297	1.97E−08
T cell (general)	CD3D	0.275	7.86E−08	0.348	3.02E−11
	CD3E	0.249	1.36E−06	0.362	4.07E−12
	CD2	0.253	8.58E−07	0.352	1.62E−11
B cell	CD19	0.232	6.58E−06	0.256	1.51E−06
	CD79A	0.249	1.20E−06	0.323	7.89E−10
Monocyte	CD86	0.279	5.30E−08	0.379	3.09E−13
	CD115 (CSF1R)	0.188	2.85E−04	0.275	2.13E−07
TAM	CCL2	0.137	8.51E−03	0.19	3.87E−04
	CD68	0.273	1.06E−07	0.331	2.76E−10
	IL10	0.23	7.82E−06	0.302	1.10E−08
M1 Macrophage	INOS (NOS2)	0.036	4.85E−01	0.052	3.36E−01
	IRF5	0.315	5.17E−10	0.314	2.59E−09
	COX2(PTGS2)	0.159	2.07E−03	0.234	1.11E−05
M2 Macrophage	CD163	0.081	1.18E−01	0.14	9.00E−03
	VSIG4	0.059	2.53E−01	0.109	4.35E−02
	MS4A4A	0.089	8.79E−02	0.161	2.79E−03
Neutrophils	CD66b(CEACAM8)	0.072	1.65E−01	0.091	8.98E−02
	CD11b (ITGAM)	0.124	1.70E−02	0.152	4.60E−03
	CCR7	0.197	1.35E−04	0.291	3.55E−08
Natural killer cell	KIR2DL1	− 0.003	9.50E−01	− 0.012	8.18E−01
	KIR2DL3	0.148	4.34E−03	0.17	1.54E−03
	KIR2DL4	0.126	1.51E−02	0.152	4.56E−03
	KIR3DL1	0.035	5.04E−01	0.049	3.63E−01
	KIR3DL2	0.149	3.99E−03	0.187	4.73E−04
	KIR3DL3	0.013	8.09E−01	0.016	7.63E−01
	KIR2DS4	0.075	1.47E−01	0.088	1.04E−01
Dendritic cell	HLA-DPB1	0.221	1.85E−05	0.297	1.91E−08
	HLA-DQB1	0.161	1.90E−03	0.226	2.23E−05
	HLA-DRA	0.171	9.37E−04	0.238	8.11E−06
	HLA-DPA1	0.185	3.48E−04	0.264	6.66E−07
	BDCA-1(CD1C)	0.291	1.19E−08	0.356	1.02E−11
	BDCA-4(NRP1)	0.352	4.11E−12	0.359	6.60E−12
	CD11c (ITGAX)	0.268	1.77E−07	0.34	9.01E−11
Th1	T-bet (TBX21)	0.161	1.91E−03	0.238	7.87E−06
	STAT4	0.19	2.42E−04	0.221	3.35E−05
	STAT1	0.339	2.56E−11	0.382	1.98E−13
	IFN-γ (IFNG)	0.217	2.47E−05	0.261	9.20E−07
	TNF-α (TNF)	0.301	3.49E−09	0.376	4.90E−13
Th2	GATA3	0.255	6.17E−07	0.343	5.58E−11
	STAT6	0.107	3.85E−02	0.106	4.95E−02
	STAT5A	0.327	1.13E−10	0.365	2.58E−12
	IL13	0.077	1.40E−01	0.066	2.25E−01
Tfh	BCL6	0.214	3.47E−05	0.208	1.01E−04
	IL21	0.086	9.91E−02	0.12	2.63E−02
Th9	TGFBR2	0.215	2.99E−05	0.244	4.43E−06
	IRF4	0.283	3.05E−08	0.366	2.38E−12
	PU.1(SPI1)	0.308	3.08E−09	0.407	3.28E−15
Th17	STAT3	0.064	2.21E−01	0.081	1.34E−01
	IL17A	0.13	1.20E−02	0.156	3.64E−03
Th22	CCR10	0.352	2.74E−12	0.359	6.31E−12
Treg	FOXP3	0.11	3.38E−02	0.15	5.35E−03
	CCR8	0.24	3.01E−06	0.292	3.28E−08
	STAT5B	0.293	9.95E−09	0.285	6.90E−08
	TGFβ (TGFB1)	0.34	2.25E−11	0.403	7.02E−15
T cell exhaustion	PD-1 (PDCD1)	0.318	3.63E−10	0.386	1.09E−13
	CTLA4	0.308	1.39E−09	0.38	2.65E−13
	LAG3	0.304	2.67E−09	0.346	4.12E−11
	TIM-3 (HAVCR2)	0.247	1.63E−06	0.343	5.67E−11
	GZMB	0.159	2.13E−03	0.199	1.95E−04

## Discussion

Liver cancer is one of the most frequent and fatal digestive malignancies and leads to over one million deaths every year around the world [33, 34]. LIHC is a highly aggressive disease and its 5-year post-surgical survival rate is 30%–40% [4, 35]. China has the highest incidence of liver cancer across the world [36]. Intrahepatic dissemination, extrahepatic infiltration and metastasis are the leading reason of poor prognosis in LIHC patients [37, 38]. The incidence of LIHC is continually increasing, Nevertheless, there is no successful therapy [39].

CPNE1, a tumor-related gene, plays the role of proto-oncogene to promote tumor development. Similar to other CPNE1 family members, the specialized structures determine the key role of CPNE1 in membranes transport and signal transduction [12]. Via vWA domain, CPNE1 could recruit, modulate transcription factors NF-kB and then activate TNF-α receptor, which in turn regulated TNF-α signaling. Meanwhile, the upregulation of TNF-α influenced the expression of CPNE1 and a positive feedback mechanism existed between CPNE1 and TNF-α. Also, CPNE1 mediated NF-kB signaling by facilitating TNF-α-dependent Inhibitory-κB (IkB) degradation [40, 41]. Via interacting directly with p65, CPNE1 lead to shear of p65 N-terminus and terminated the transcription of NF-kB, which in turn inhibited transcriptional activation of NF-kB [42]. It had been recognized that NF-kB was linked to multiple behaviors of cancer cells, including cell proliferation, apoptosis, migration and invasion [43] and played pivotal functions in initiation and progression of many malignancies (breast cancer, lung cancer, gastric cancer) [44–47]. Study had reported that CPNE1 could promote proliferation and multi-differentiation potency of neuronal stem cells by activating AKT/mTOR signaling [48]. CPNE1 may regulate growth, migration and invasion of lung adenocarcinoma cells through AKT and ERK pathways, which could promote nonsmall-cell lung cancer progression [16]. CPNE1 was a target of miR-335–5 and CPNE1 silencing could effectively improve clinical responses of EGFR-tyrosine kinase inhibitors (TKIs) in non-small cell lung cancer [49]. In osteosarcoma, downregulation of CPNE1 not only significantly impaired the proliferation and metastasis of Saos-2 cell and enhanced sensitivity to cisplatin and doxorubicin, but also changed the expression of genes related to ECM receptors-associated pathway, MAPK pathway, TGF-β pathway, apoptotic pathway and NOD-like receptor pathway [20]. CPNE1 may promote tumorigenesis and radioresistance of triple-negative breast cancer (TNBC) through AKT pathway activation and so target expression of CPNE1 could be a good strategy to sensitize TNBC to radiotherapy [15]. But the role of CPNE1 in liver cancer is not clear.

The role of a great deal genes is complex in the human body. The development of bioinformatics can markedly improve the accuracy and efficiency of studies target genes and cancer [50, 51]. In our study, we confirmed the expression of CPNE1 was higher in LIHC tissue than that in normal tissues. High expression of CPNE1 showed potential clinical significance and was linked to poor survival of LIHC patients. These results indicated that CPNE1 was a potential target for LIHC treatment. To explore the intrinsic mechanisms of CPNE1 in LIHC, the coexpression network of CPNE1 was constructed and gene set enrichment analysis demonstrated CPNE1 and its coexpressed genes were primarily involved in the establishment of protein localization to membrane, ribonucleoprotein complex biogenesis, lipid localization and response to xenobiotic stimulus. KEGG results showed these coexpressed genes were mainly enriched in ribosome, spliceosome and metabolic pathways. CPNE1 contained 1 VWA domain structure. The VWA domain is a common domain involved in cell adhesion, in extracellular matrix proteins, and in integrin receptors. Most of VWA-containing proteins involved in functions such as transcription, membrane transport, ribosomal, DNA repair, and the proteasome [52]. Through the VWA domain, CPNE1 and its coexpressed genes may be linked to ribosome, spliceosome and metabolic pathways.

For exploring regulators potentially responsible for CPNE1 dysregulation, we found that CPNE1 is linked with a network of kinases including ATR, CHEK1, PLK3, CHEK2 and DAPK1 in LIHC. These kinases participate in the regulation of mitosis, DNA damage response, cell cycle and genomic stability, and exhibited survival prognosis and differential expression in LIHC. In fact, ATR, a member of phosphatidylinositol-3-kinase-related kinase family, is the major players of DNA damage response, and represents an attractive target for developing antimitotic agents [53]. In addition, activated ATR is critical in the late G2 and S phases to assure appropriate and replication of the whole genome [54, 55]. PLK3 may regulate cell cycle progression, centrosomal functions, mitosis, DNA replication, and Golgi fragmentation [56]. In many human malignancies, PLK3 expression was downregulated, including those in the stomach, kidney, head and neck, lung, colon, liver and rectum. Several studies demonstrated downregulated PLK3 expression may be linked with cancer development [55, 56].

MIR-493 and MIR-381 were the mainly miRNA Targets of CPNE1 in LIHC. MiR-493 has important functions and participates in different oncogenesis, including breast cancer, pancreatic cancer and gastric cancer. MiR-493 plays a key role in the recurrence, metastasis and generation of tumors [57]. MiR-381, one of the most significant miRNAs, regulates radioresistance [58], immune responses [59], epithelial–mesenchymal transition (EMT) [60] and chemotherapeutic resistance [61]. Moreover, miR-381 functions in AKT [62], p53 [63] and Wnt/β-catenin [64] pathways, involves in tumor metastasis, progression and initiation. Multiple studies have indicated that miR-381 could be recognized as a biomarker [65].

Then, the E2F family account for the main transcription factors for CPNE1 dysregulation. E2F1 is one of the major bonds in the cell cycle regulatory network. In the progression of LIHC, activated E2F signaling was common, and studies have indicated that dosage-dependent copy number gains in E2F3 and E2F1 drive LIHC [66]. Our findings indicate that E2F1 is a critical regulator of CPNE1 and that CPNE1 might function by this factor to modulate the proliferation ability and cell cycle of LIHC.

Here, we revealed that the overexpression of CPNE1 was positively linked to immune infiltration. This finding demonstrates that CPNE1 plays a crucial role in immune infiltration during hepatocarcinogenesis. As far as we know, we are the first to explore the association of CPNE1 and immune infiltration in LIHC.

To validate the effect of CPNE1 on cell proliferation, migration and invasion in LIHC cell lines, we constructed CPNE1 knockdown and overexpression cell lines and results revealed that CPNE1 participated in the genesis and progression of LIHC. Furthermore, CPNE1 affected AKT/P53 pathway and might function by this pathway to modulate the malignant transformation of LIHC.

Previous studies have shown CPNE1 is upregulated in multiple tumor types. And CPNE1 participates in the process of carcinogenesis and development of different tumors and is associated with TNM staging, metastasis and prognosis of multiple carcinomas. It has mentioned that CPNE1 mediates the process of progression by regulating the dedifferentiation, cell cycle and proliferation in liver cancer. But the mechanisms underlying these phenomena have not been clearly explained. In this study, we focus on the specific role of CPNE1 in LIHC and explore the underlying mechanisms. Our study firstly authenticated the expression of CPNE1 was significantly higher in LIHC tissues than in normal liver tissues, and high CPNE1 expression was associated with poor prognosis. In addition, we firstly identified the mechanism by which CPNE1 functions in LIHC. CPNE1 influenced AKT/P53 pathway activation and LIHC cell proliferation and migration. There was a significant correlation between CPNE1 expression and tumor immune infiltration in LIHC. However, this study had some limitations. First, our findings were just confirmed in public databases and not in our own clinical samples. Second, although we suggested that CPNE1 could affect AKT/P53 signaling, the precise regulatory mechanism of CPNE1 involved in the development of LIHC needs further in-depth study. Last, more experiments should be done to further investigate the effects of CPNE1 on tumor immune infiltration.

## Conclusions

The expression of CPNE1 was significantly higher in LIHC tissues than in normal liver tissues, and high CPNE1 expression was associated with poor prognosis. CPNE1 influenced the biological behaviors of LIHC cells and regulated AKT/P53 pathway activation in LHC. There was also a significant correlation between CPNE1 expression and tumor immune infiltration in HCC. On the whole, CPNE1 is a promising molecular target for the therapy of LIHC.

## Supplementary Information

**Additional file 1. **The densitometry analysis of CPNE1 for western blots in LIHC cell lines transfected CPNE1-siRNA. (a) HepG2. (b) MHCC-97H.

**Additional file 2. **The AKT pathway was enriched in high-risk group by GSEA.

**Additional file 3. **The densitometry analysis of p-AKT and P53 for western blots in LIHC cell lines transfected CPNE1-siRNA. (a, b) HepG2. (c, d) MHCC-97H.

**Additional file 4. **The densitometry analysis of CPNE1 for western blots in Hep3B transfected CPNE1 overexpression plasmid.

**Additional file 5. **The densitometry analysis of p-AKT and P53 for western blots in Hep3B transfected CPNE1 overexpression plasmid. (a) p-AKT. (b) P53.

**Additional file 6.** The sequence of CPNE1 overexpression plasmid.

## Data Availability

The data used to support the findings of this study are included within the manuscript.
