# Supplementary figures and images for: CPNE1 is a potential prognostic biomarker, associated with immune infiltrates and promotes progression of hepatocellular carcinoma
Source: Cancer Cell Int. 2022 Feb 9;22:67. doi: 10.1186/s12935-022-02485-2 (PMC8826718; doi:10.1186/s12935-022-02485-2)

Figure S2.


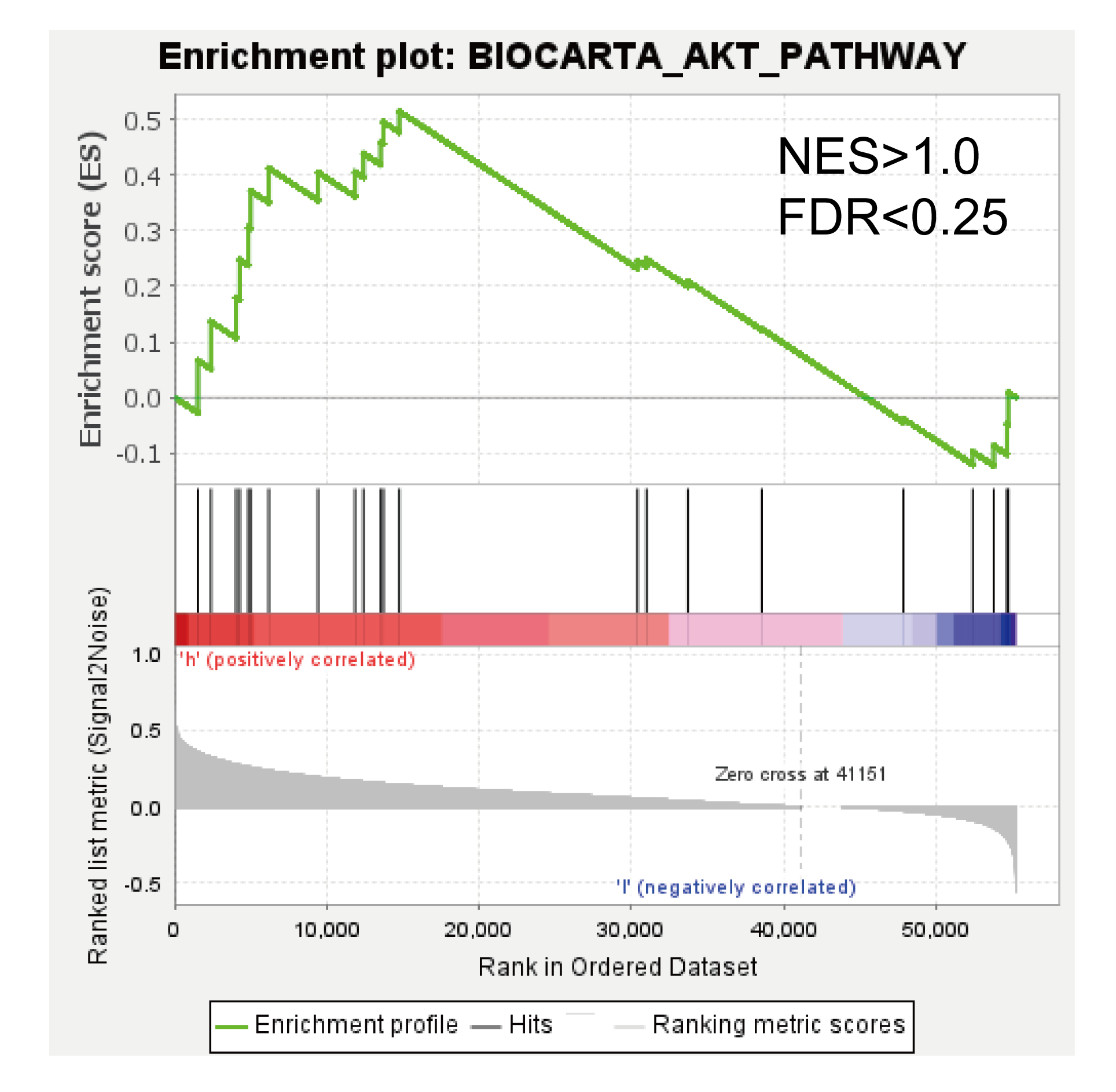


The AKT pathway was enriched in high-risk group by GSEA.

Supplement: Supplementary file 2 — Additional file 2. The AKT pathway was enriched in high-risk group by GSEA. [file 12935_2022_2485_MOESM2_ESM.docx]
