# Supplementary material for: CPNE1 is a potential prognostic biomarker, associated with immune infiltrates and promotes progression of hepatocellular carcinoma
Source: Cancer Cell Int. 2022 Feb 9;22:67. doi: 10.1186/s12935-022-02485-2 (PMC8826718; doi:10.1186/s12935-022-02485-2)
Supplement: Supplementary file 3 — Additional file 3. The densitometry analysis of p-AKT and P53 for western blots in LIHC cell lines transfected CPNE1-siRNA. (a, b) HepG2. (c, d) MHCC-97H. [file 12935_2022_2485_MOESM3_ESM.docx]

Figure S3.


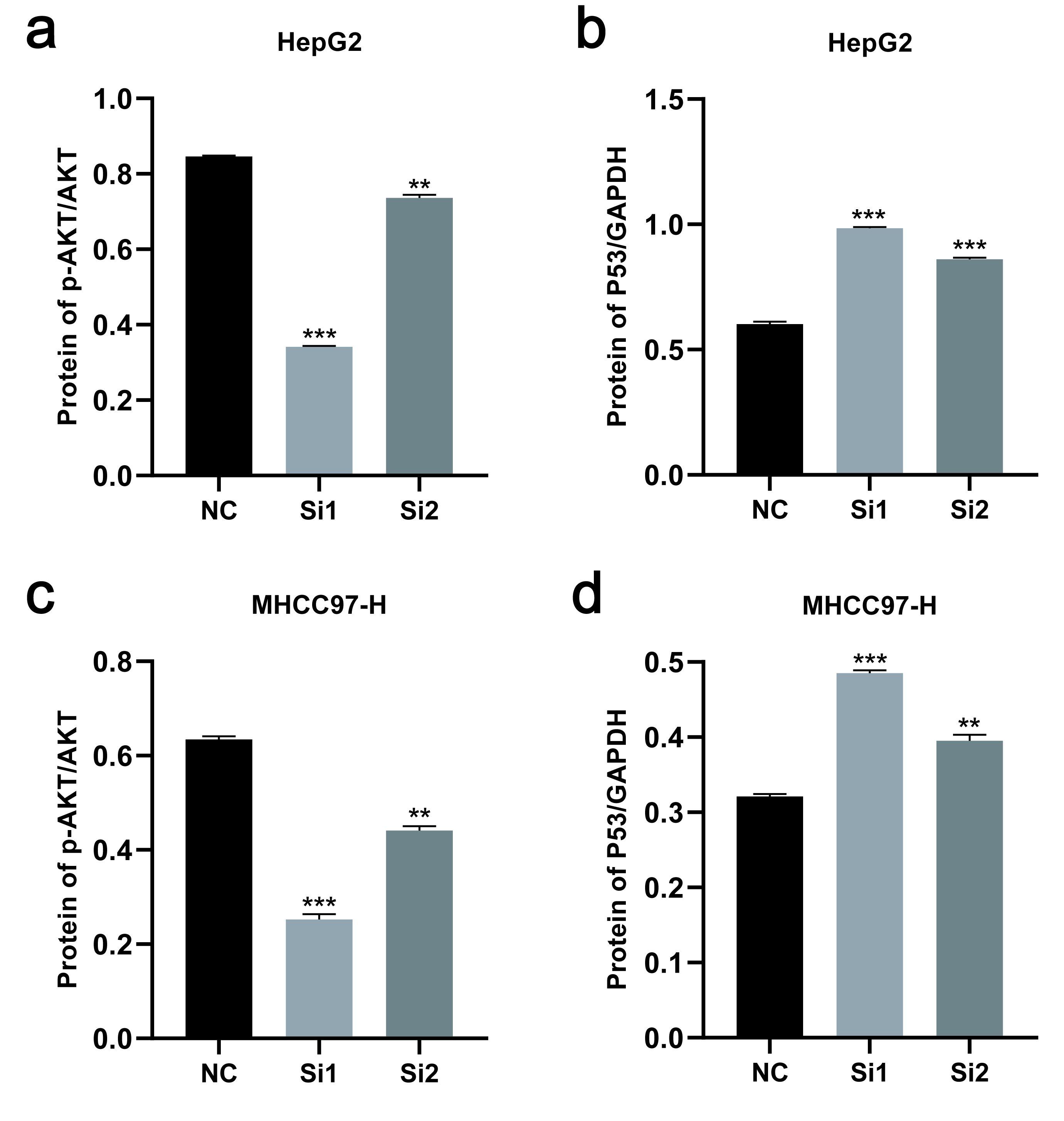


The densitometry analysis of p-AKT and P53 for western blots in LIHC cell lines transfected CPNE1-siRNA. (a, b) HepG2. (c, d) MHCC-97H.
