# Supplementary material for: CPNE1 is a potential prognostic biomarker, associated with immune infiltrates and promotes progression of hepatocellular carcinoma
Source: Cancer Cell Int. 2022 Feb 9;22:67. doi: 10.1186/s12935-022-02485-2 (PMC8826718; doi:10.1186/s12935-022-02485-2)
Supplement: Supplementary file 4 — Additional file 4. The densitometry analysis of CPNE1 for western blots in Hep3B transfected CPNE1 overexpression plasmid. [file 12935_2022_2485_MOESM4_ESM.docx]

Figure S4.


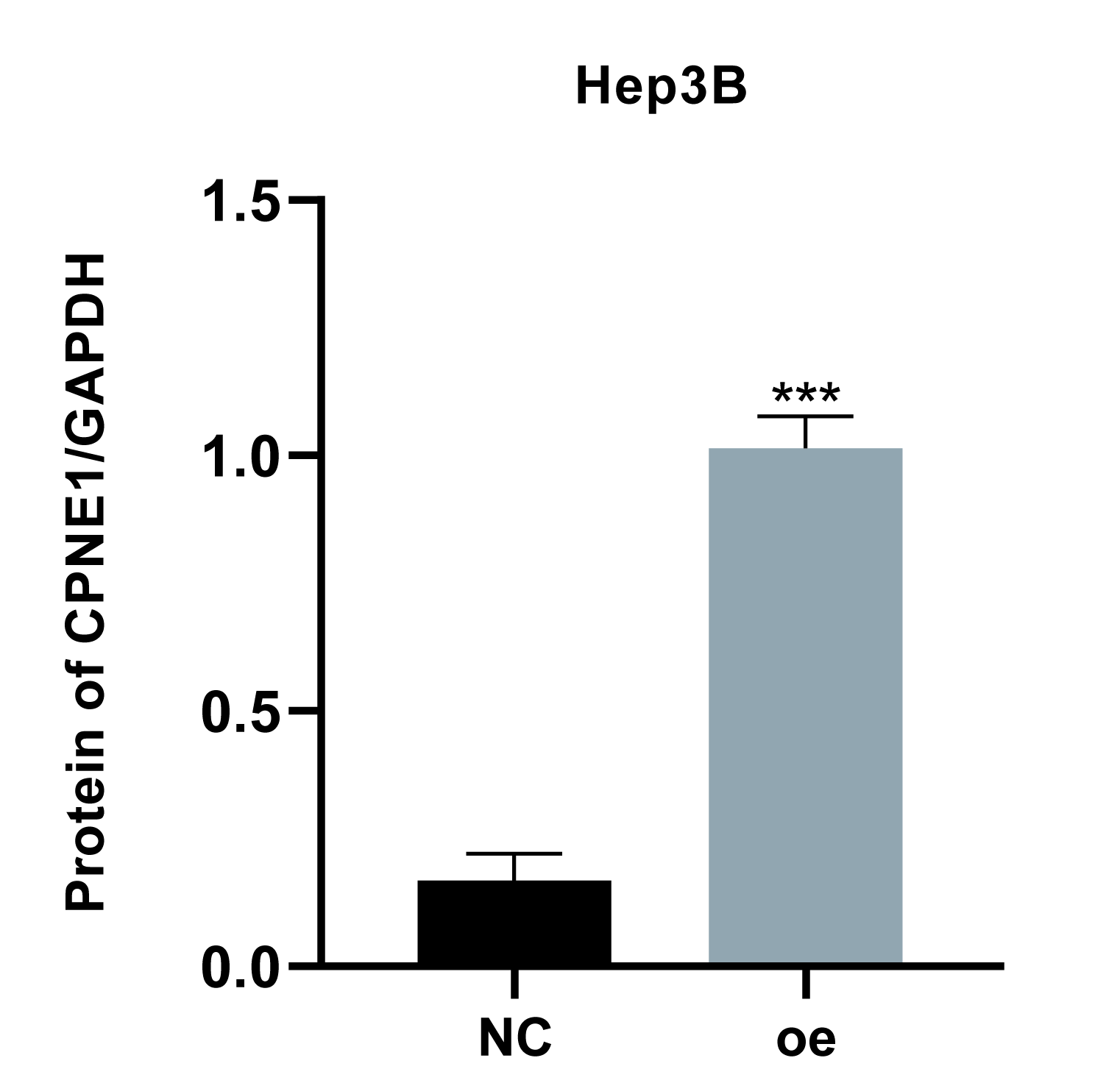


The densitometry analysis of CPNE1 for western blots in Hep3B transfected CPNE1 overexpression plasmid.
